# Metabolomics for biomarker discovery in the diagnosis, prognosis, survival and recurrence of colorectal cancer: a systematic review

**DOI:** 10.18632/oncotarget.16727

**Published:** 2017-03-30

**Authors:** Fan Zhang, Yuanyuan Zhang, Weiwei Zhao, Kui Deng, Zhuozhong Wang, Chunyan Yang, Libing Ma, Margarita S. Openkova, Yan Hou, Kang Li

**Affiliations:** ^1^ Department of Epidemiology and Biostatistics, School of Public Health, Harbin Medical University, Harbin, P.R. China; ^2^ Harbin Medical University, Harbin, P.R. China

**Keywords:** systematic review, metabolomics, CRC, biomarkers, pathway

## Abstract

Colorectal cancer (CRC) remains an incurable disease. There are no effective noninvasive techniques that have achieved colorectal cancer (CRC) diagnosis, prognosis, survival and recurrence in clinic. To investigate colorectal cancer metabolism, we perform an electronic literature search, from 1998 to January 2016, for studies evaluating the metabolomic profile of patients with CRC regarding the diagnosis, recurrence, prognosis/survival, and systematically review the twenty-three literatures included. QUADOMICS tool was used to assess the quality of them. We highlighted the metabolism perturbations based on metabolites and pathway. Metabolites related to cellular respiration, carbohydrate, lipid, protein and nucleotide metabolism were significantly altered in CRC. Altered metabolites were also related to prognosis, survival and recurrence of CRC. This review could represent the most comprehensive information and summary about CRC metabolism to date. It certificates that metabolomics had great potential on both discovering clinical biomarkers and elucidating previously unknown mechanisms of CRC pathogenesis.

## INTRODUCTION

Colorectal cancer (CRC) is the third most common type of cancer and the fourth leading cause of cancer-related deaths worldwide [1]. In China, the crude mortality rate for CRC ranks fifth in cancer-related deaths in all cancer sites with a rate of 11.11/100,000, and the estimate of new diagnosed cases in 2011 was 310,244, accounting for 9.20% of overall new cancer cases [2, 3]. The early diagnosis of CRC is critical. If patients with CRC were diagnosed in the early stage, the 5-year survival rate could have been up to 90%. Unfortunately, more than 60% of CRC cases had already developed to an advanced stage by the time of detection, resulting in a survival rate around 8-9% [4, 5]. Although, the pre-operative endoscopic and radiological imaging has been used for CRC diagnosis, these invasive techniques suffer from poor patient compliance [6]. Currently, noninvasive monitoring tests, e.g. fecal occult blood test (FOBT) and tumor markers, including carcinoembryonic antigen (CEA) and carbohydrate antigen 19-9 (CA19-9), have been commonly used in clinical settings. However, unsatisfactory sensitivity and specificity have limited the clinical application in CRC diagnosis, prognosis and survival significantly [7]. Therefore, it is urgent and important to develop noninvasive and accurate screening tools to facilitate early detection and precise staging of CRC. So far, the metabolomics biomarkers have been considered a promising approach to discover the potential biomarkers for monitoring the tumor progression, regression and recurrence, further ensuring that all patients receive the proper treatment.

Metabolomics, as the endpoint of the ‘omics’ cascade, focuses on investigating the global metabolites presented in a biological specimen. Currently, it has been widely used to investigate its potential in biomarker discovery for diagnosis, treatment, and prevention, based on individual cancers. Some studies have been conducted to summarize these metabolites across different studies, based on specific aim, e.g. diagnosis or from analytic platform [7–10]. For example, Zhang et al. reviewed the potential role of small molecule metabolites in cancer research and highlighted some metabolomic publications on CRC [8]. Ni et al. focused on the recent advances and findings in the biomarker discovery for the early diagnosis and prognosis in CRC, based on different analytic platforms [7]. Armitage et al. focused on the approaches in metabolomics that have been used in cancer biomarker discovery and further research in this field [10]. Although, previous studies have been performed to summarize the potential biomarkers for CRC diagnosis, these studies have been performed on some metabolomic journals, rather than all journals. Moreover, these studies have not been conducted to further investigate the metabolite classes and pathway-related dysfunctions in CRC diagnosis, recurrence, prognosis and survival, especially comparing the metabolites across studies to observe whether these metabolites could be replicated across studies.

In our study, we highlighted the metabolism perturbations based on metabolites and pathways across CRC metabolomic publications. Furthermore, the metabolite concentrations in the CRC patients were compared with controls across different studies to observe whether the change trends were consistent, regardless of the heterogeneity of patients and controls. These results would support further studies on validating these metabolites and exploring the possible metabolic pathways in CRC.

## RESULTS

### Searching process

**Figure 1:**
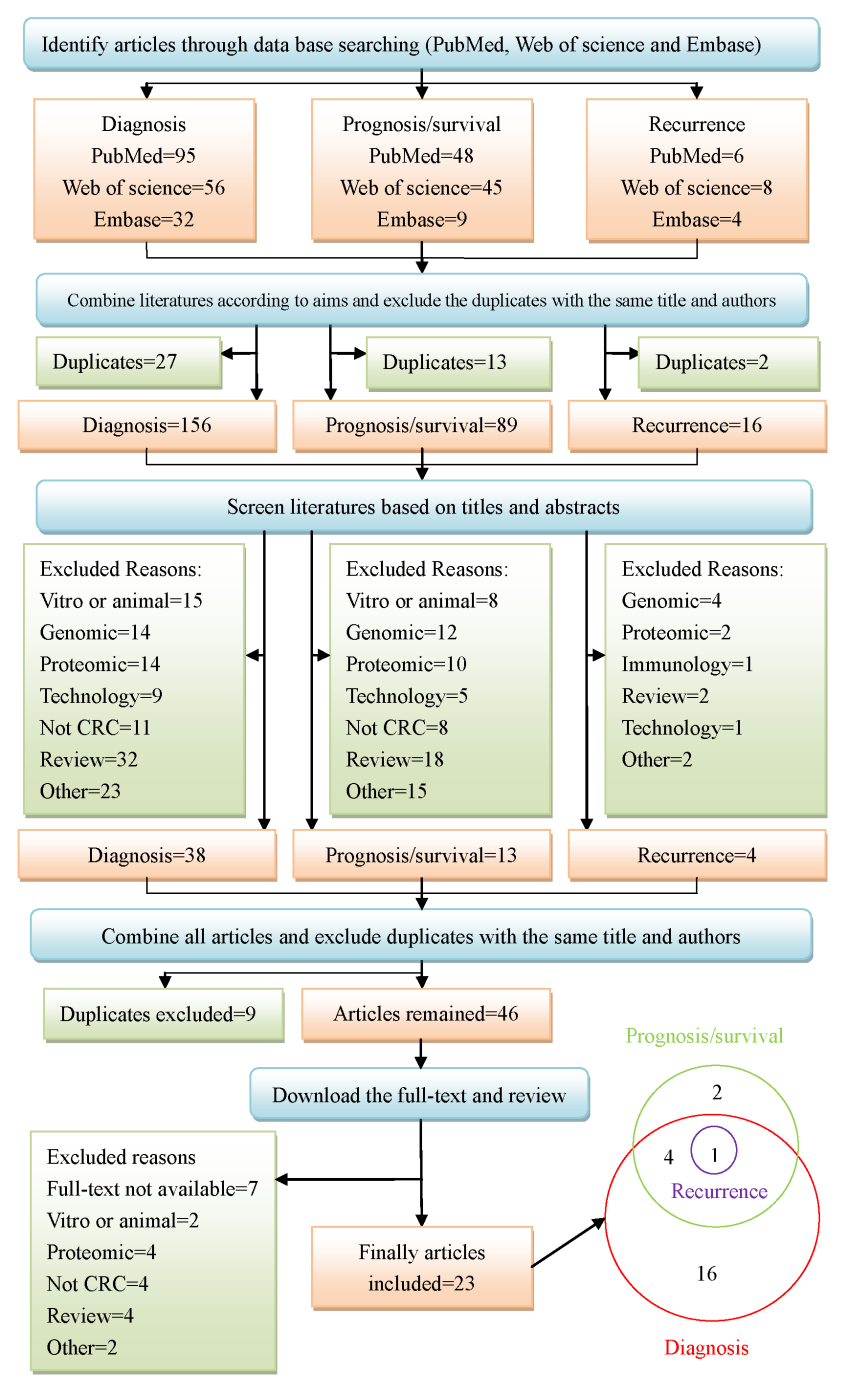
Systematic search and selection strategy

The working flow diagram was displayed in Figure 1. When we searched three databases with the combination of the keywords mentioned above, ninety-five, fifty-six, and thirty-two studies were selected for diagnosis from PubMed, Web of Science and Embase, separately. Forty-eight, forty-five, and nine studies were selected for prognosis or survival, separately. Six, eight, and four studies were selected for recurrence, separately. We combined databases corresponding to each aim and excluded duplicates. One hundred and fifty-six studies remained for diagnosis, eighty-nine for prognosis or survival, and sixteen for recurrence. Then we screened the literature based on title and abstract. Thirty-eight studies remained for diagnosis, thirteen for prognosis or survival, and four for recurrence. At last, we combined all articles and excluded duplicates. Forty-six studies were further acquired to access full-text. Unfortunately, seven studies were without full-text. Therefore, thirty-nine full text studies were reviewed in detail, and sixteen studies were excluded due to different reasons, which were presented in Figure 1. Twenty-three studies were finally eligible for systematic review, of which sixteen studies were about diagnosis, two studies on prognosis or survival, four studies on diagnosis, prognosis or survival, and one on diagnosis, prognosis, survival and recurrence.

### Quality assessment

The quality assessment results, in accordance with the QUADOMICS tool, were shown in Supplementary Table S1. According to the quality assessment, 10 (43%) of the studies were not able to avoid over-fitting due to lack of an independent validation set. 19 (83%) of the studies were prospective researches. All the studies included in this review were explorative. Thus, items questioning the availability of the clinical data and the representative nature of the spectrum of patients, when a metabolomic platform was used in practice, were not applicable for all the studies included. The detailed questioning items for all studies were shown in Supplementary Table S1.

### Study characteristics

**Figure 2:**
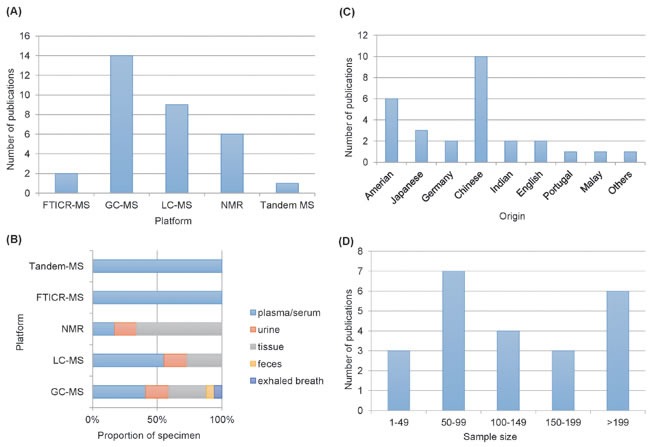
**A**. Comparison of analytical platforms in CRC metabonomics. **B**. Proportion of biological samples in platform. **C**. comparison of organics in CRC metabonomics. **D**. Sample size in different metabonomics studies.

Biological samples utilized for metabolomic analysis included serum/plasma in 11 studies, urine in 4 studies, tissue in 9 studies, exhaled breath in 1 study, and feces in 1 study, where both plasma and tissue were included in 2 studies, and both feces and tissue were used in 1 study. The analytical platforms, used for metabolite detection, included liquid chromatography mass spectrometry (LC-MS) in 9 studies, gas chromatography mass spectrometry (GC–MS) in 14 studies, nuclear magnetic resonance (NMR) in 6 studies, Fourier transform ion cyclotron resonance mass spectrometry (FTICR-MS) in 2 studies and tandem MS in one study (Figure 2A. The platforms of publications, the proportion of the specimen in platforms, the year of publications, the sample size and the origin of the publications are shown in Figure 2. The first author's name, publication year, specimen type, study group, sample size, platform, origin and the main aim of the articles are summarized in Table 1. Detailed regulation of metabolites according to related pathways is presented in the Table 2 and electronic supplementary materials (Supplementary Tables S2, S3, S4 and S5).

**Table 1:** Current literature in metabolomics of colorectal cancer detection

No	Ref	Specimen	Cases/controls	Platform	Origin	Aim
**1**	Cross et al., 2014 [48]	Serum	CRC (*n* = 254); Match control nested in other cancer (*n* = 254)	UPLC-MS; GC-MS	Amerian	Diagnosis
**2**	Ikeda et al., 2012 [25]	Serum	Esophageal (n = 12); Gastric (*n* = 11); CRC (*n* = 16); Healthy control (*n* = 12)	GC-MS	Japanese	Diagnosis
**3**	Leichtle et al., 2012 [26]	Serum	CRC (*n* = 59); Healthy control (*n* = 58)	Tandem-MS	Germany	Diagnosis
**4**	Li et al., 2013 [12]	Serum	CRC (n = 52); Healthy control (n = 52)	DI-ESI(±)-FTICR-MS	Chinese	Diagnosis
**5**	Nishiumi et al., 2012 [49]	Serum	CRC (*n* = 60); Matched healthy control (*n* = 60)	GC-MS	Japanese	Diagnosis
**6**	Ma et al., 2012 [22]	Serum	CRC (*n* = 30); Healthy control (*n* = 30)	GC-MS	Chinese	Diagnosis
**7**	Ritchie et al., 2010 [50]	Serum	CRC and healthy control from three independent populations (*n* = 222)	HPLC-MS; NMR FTICR-MS	American; Japanese	Diagnosis
**8**	Tan et al., 2013 [27]	Serum	CRC (*n* = 101); Healthy control (*n* = 102)	GC−TOF-MS UPLC-QTOF-MS	Chinese	Diagnosis
**9**	Zhu et al., 2014 [14]	Serum	CRC (*n* = 66); Polyp control (*n* = 76); Healthy control (*n* = 92)	LC-MS-MS	Indianan	Diagnosis
**10**	Manna et al., 2014 [28]	Tissue	CRC mucosa (*n* = 39); Normal mucosa (*n* = 39)	UPLC-MS	American	Diagnosis
**11**	Mirnezami et al., 2014 [16]	Tissue	CRC mucosa (*n* = 44); Normal mucosa (*n* = 44)	HR-MAS-NMR	English	Diagnosis
**12**	Wang et al., 2013[13]	Tissue	CRC mucosa (*n* = 127); Normal mucosa (*n* = 43)	1H-NMR	Chinese	Diagnosis
**13**	Silva et al., 2011[51]	Urine	CRC (*n* = 33); Healthy control (*n* = 21)	GC-MS	Portugal	Diagnosis
**14**	Wang et al., 2014[52]	Exhaled breath	CRC (*n* = 20); Healthy control (*n* = 20)	GC-MS	Chinese	Diagnosis
**15**	Liesenfeld et al., 2015[21]	Serum; Tissue	Visceral adipose tissue (*n* = 59); Subcutaneous adipose tissue (*n* = 59)	GC-MS; LC-MS	Germany	Diagnosis
**16**	Dowling et al., 2015[53]	Plasma; Tissue	CRC (*n* = 56); Healthy control (*n* = 30)	UHPLC-MS-MS; GC-MS	American	Diagnosis
**17**	Liesenfeld et al., 2015[15]	Urine	CRC prior to surgery (*n* = 97); 1-8days post-surgery (*n* = 12); 6 months follow-up (*n* = 52); 12 months follow-up (*n* = 38)	GC-MS; 1H-NMR	American	Prognosis/ Survival
**18**	Phua et al., 2014[23]	Tissue; Feces	CRC (*n* = 11); Healthy control (*n* = 10)	GC-TOF-MS	Chinese	Prognosis/ Survival
**19**	Chan et al., 2009[19]	Tissue	CRC mucosa (*n* = 32); Normal mucosa(*n* = 31)	HR-MAS-NMR; GC-MS	Chinese; Indian; Malay; Other ethnicity	Diagnosis; Prognosis/ Survival
**20**	Jiménez et al., 2013[17]	Tissue	CRC mucosa (*n*= 82); Normal mucosa (*n* = 87)	HR-MAS-NMR	English	Diagnosis; Prognosis/ Survival
**21**	Cheng et al., 2012[11]	Urine	CRC (*n* = 101); Healthy control (*n* = 103)	GC-TOF-MS; UPLC-QTOF-MS	Chinese	Diagnosis; Prognosis/ Survival
**22**	Yue et al., 2013[54]	Urine	CRC (*n* = 29); Healthy control (*n* = 10)	RRLC-QTOF-MS	Chinese	Diagnosis; Prognosis/ Survival
**23**	Qiu et al., 2014[18]	Tissue	Surgical specimens from four CRC patient cohorts (*n* = 376)	GC-TOF-MS	Chinese; American	Diagnosis; recurrence; Prognosis/ Survival

**Table 2:** The information of the most important biomarkers based on applications in clinical

Applicaiton	Marker	Fold change^$^	P-value^$^	VIP^$^	N*	Perturbation^&^	Type^#^
Key change^a^	Arabitol	−1.82	-	-	2	decreasing	S2
Key change	Galactose	−36.8	<0.0005	-	2	decreasing	S2
Key change	Mannose	-	<0.05	-	2	decreasing	S2
Key change	pyruvate	−1.15	0.0307	-	2	decreasing	S2
Key change	Hydroxybutyrate	1.95	0.0008	1.28	2	increasing	S3
Key change	Glycochenodeoxycholate	1.42	<0.005	-	2	increasing	S3
Key change	1-Octanol	-	-	-	2	decreasing	S3
Key change	Phosphocholine (PC)	-	<0.01	1.68	2	increasing	S3
Key change	Oleic acid	1.5	0.0009	1.58	2	increasing	S3
Key change	Glutamic acid	-	-	-	2	increasing	S4
Key change	Histidine	−1.24±0.06	<0.005	1.91	3	decreasing	S4
Key change	Iso-glutamine	1.7	-	-	2	increasing	S4
Key change	Methionine	−1.15±0.04	<0.0001	1.30	3	decreasing	S4
Key change	Tryptophan	−1.52±0.10	<0.0001	1.63±0.75	2	decreasing	S4
Key change	Phenol	−2.99±0.20	<0.0001	1.72±0.78	2	decreasing	S5
Key change	Carnitine	1.23	0.00007	1.25	2	increasing	S5
Key change	Urea	−1.39±0.03	<0.0001	1.44±0.20	2	decreasing	S5
Stage	Glucose	-	<0.05	-	6	decreasing	S2
Stage	Succinate	1.84	<0.001	2.14	2	increasing	S2, S3
Stage	GPC	-	<0.005	-	1	increasing	S3
Stage	Triglycerides	−1.3	<0.005	-	2	decreasing	S3
Stage	Fumarate	(−,1.81)	<0.005	1.30	2	contradictory	S3
Stage	Taurine	(−2.10,1.3)^b^	(<0.001, <0.0005)	(4.25, -)	4	contradictory(1:3)	S4
Stage	Tyrosine	(1.56,1.3±0.08)	(<0.001,<0.001)	(1.42, -)	4	contradictory(2:2)	S4
Stage	Phenylalanine/L-Phenylalanine	(−1.35, 1.3)	(<0.0001,-)	(1.98,-)	4	contradictory(2:2)	S4
Stage	P-cresol	(−3.57,- )	-	(1.05,- )	2	contradictory	S5
Stage	Kynurenate	−2.50	<0.005	2.17	1	decreasing	S5
Early diagnosis	Hexadecanedioic acid	−1.4	-	-	1	decreasing	S3
Early diagnosis	LPC(20:4)	1.7	-	-	1	increasing	S3
Early diagnosis	LPC(22:6)	1.3	-	-	1	increasing	S3
Early diagnosis	LPC(16:0)	1.4	-	-	1	increasing	S3
Early diagnosis	Octadecanoic acid	−1.5	-	-	1	decreasing	S3
Early diagnosis	Palmitic amide	−2.4	-	-	1	decreasing	S3
Recurrence	Palmitoleate	2.25	-	1.90	1	increasing	S3
Recurrence	Uracil	(1.59,2.9±1.25)	(<0.001, <0.003)	(1.15,1.56±0.53)	5	contradictory(4:1)	S5
Recurrence	Lactate	1.33±0.25	<0.01	2.1±0.33	5	increasing	S2, S3
Recurrence	Glycerol	1.48	0.0003	1.36	1	increasing	S2
Recurrence	Myoinositol	−1.29	0.008	1.10	3	decreasing	S3
Recurrence	Myristate	(− ,1.72)	( ,0.00006)	( ,1.56)	2	contradictory	S3
Recurrence	5-Oxoproline	1.76	<0.005	1.96	1	increasing	S4
Recurrence	Aspartate	1.70	0.01	1.92	1	increasing	S4
Recurrence	cysteine	1.62	<0.005	1.64	1	increasing	S4
Recurrence	Alanine/ L-Alanine/β-Alanine	(−1.29±0.16, 2.99±2.22)	-	(1.3±0.42,2.48)	6	contradictory(4:2)	S4
Recurrence	Glutamate	(−1.29,-)	(<0.0001, -)	(1.02,-)	2	contradictory	S4
Recurrence	Kyrunine	4.31	<0.0001	2.55	1	increasing	S5
Recurrence	Hypoxanthine	1.38	0.03	1.84	2	increasing	S5
Prognosis/Survival	Choline	1.2	-	-	2	increasing	S3
Recurrence and Stage	2-Aminobutyrate	1.62±0.18	-	1.8±0.45	2	increasing	S3
Recurrence and Survival	Iso-butyrate	1.4	-	-	1	increasing	S3
Recurrence and Stage	Acetate	2.97	<0.005	2.27	2	increasing	S3
Recurrence and Stage	Putrescine	1.53±0.08	<0.005	1.1±0.05	2	increasing	S4
Survival and Stage	Proline/ L-Proline	1.279	<0.01	-	5	increasing	S4
Survival and Stage	P-cresol-b-Oglucuronide	-	-	-	1	increasing	S5

Note: ^a^key change means the metabolites have the same tendency in more than one literatures.

*N means the times of biomarkers reported in literatures.

^&^increasing/decreasing=up-regulated / down-regulated in CRC.

^$^fold change, VIP of the metabolite reported in more than one literature were denoted by mean±sd. p value of the metabolite reported in more than one literature were denoted by the max one.

^b^For contradictory (A, B), A means value for the contradictory marker with down-regulated, while B for up-regulated;

-means the values were not reported

Type ^#^ means the biomarkers are from original supplementary tables, e.g Type^#^-S3 means from Table S3.

### Biomarkers related to early diagnosis and clinical staging

A systematic review of literature revealed 16 studies evaluating metabolomic biomarkers referred to early stage CRC, of which 4 studies were particularly designed for early diagnosis of CRC when compared with controls, and metabolomic profiling of different groups could be significantly discriminated from different platforms. For example, Cheng et al. performed a large-scale study to compare the urinary samples of CRC cases (n=101) with healthy controls (n=103) using ultra performance liquid chromatography quadrupole time-of-flight mass spectrometry (UPLC-QTOF-MS) and gas chromatography time-of-flight mass spectrometry (GC-TOF-MS). A principle component analysis (PCA) plot was constructed with satisfactory discriminating ability using the 261 annotated metabolites, and all of the cancer patients were correctly discriminated from the healthy controls, including 24 patients at tumor node metastasis (TNM) stage I [11]. Li et al. used FTICR-MS approach to evaluate the early diagnosis and progression with serum lipid metabolites in 52 CRC patients and 52 healthy controls. Identified biomarkers contained palmitic amide, oleamide, hexadecanedioic acid, octadecanoic acid, eicosatrienoic acid, LPC(18:2), LPC(20:4), LPC(22:6), myristic acid and LPC(16:0) [12]. Wang et al. compared CRC (n=127) and normal controls (n=43) with tissue metabolites from^1^H NMR platform [13]. Zhu et al. compared CRC cases (n=66) with polyp patients (n=76) and healthy controls (n=92), based on serum using a targeted LC-MS approach, and found that all stages of CRC, including stage I, were discriminated perfectly from controls with area under curves (AUCs) greater than 0.93 [14].

However, there were 3 studies discriminating between different stages of CRC. Liesenfeld et al. divided urine samples from CRC patients prior to surgery (n=97) into three groups: “early” meaning carcinoma in situ and localized; ‘‘intermediate’’ meaning locally advanced and locally advanced with lymph nodes affected, and ‘‘late’’ meaning metastasized. The conclusion is that early-stage patients were easier to distinguish from more advanced stages of the disease, whereas, intermediate stages were poorly differentiated from either of these groups [15]. Mirnezami et al. fitted OPLS-DA models with T1/2, T3 and T4 of CRC tissue metabolites. The metabolite-driven means of determining local tumor stage were able to correctly assign samples as T1/2, T3, or T4 in 91%, 90%, and 75% of cases, respectively. Furthermore, the approach revealed specific metabolic phenotypes associated with each stage of local tumor development [16]. Interestingly, Jiménez et al. not only classified tumor tissues according to clinical tumor-classification (T-classification) and node-classification(N-classification) of CRC, but also classified adjacent tumor mucosa according to the two classifications of CRC. Both tumor tissues and non-tumor ones could discriminate stages of CRC according to T-classification and N-classification. The results indicated that it was valuable to analyze not only tumor tissue, but also the tissue surrounding the cancerous area in terms of tumor classification, which was called “field-effects” [17]. The biomarkers, related to early diagnosis and stages, are shown in the Table 2 and electronic supplementary materials with special markers (Supplementary Tables S2, S3, S4 and S5).

### Biomarkers for recurrence, prognosis, or survival

All three studies were on diagnosis, prognosis or survival, while one study fulfilled all search aims. For example, Qiu et al. performed a large research on four independent cohorts to identify replicate biomarkers related to CRC and predict the rate of recurrence and survival for patients after surgery and chemotherapy. Finally, fifteen biomarkers were significantly and consistently altered with the same up and down tendency in all batches. A binary logistic regression analysis was then performed using recurrence results as the dichotomous-dependent variable and these 15 differential metabolites, plus age and gender, as the covariates. The AUC value for recurrence was 0.895 (95% confidence level, 0.824-0.966), with a sensitivity of 0.750, and a specificity of 0.894. Similarly, the same analysis was performed on survival results and the AUC value for survival reached 0.860 (95% confidence level, 0.771-0.949), with a sensitivity of 0.938 and a specificity of 0.746 [18]. Chan et al. performed a study which not only discriminated malignant mucosae from normal, but could also distinguish between the anatomical and clinic pathological characteristics. The anatomical and clinic pathological characteristics were closely related to prognosis [19]. A study by Jimenez et al. was performed using high-resolution magic angle spinning nuclear magnetic resonance (HR-MAS NMR) spectroscopy, analyzed metabolites in intact tumor samples (n= 83) and samples of adjacent mucosa (n= 87). The AUC of the OPLS model reached 0.91. Moreover, it used tumor and non-tumor tissue to predict cancer-specific survival, based on metabolite profiles from 5-year follow up data, respectively. The conclusion was that tumor tissue from patients with a 5-years survival and from those, who died owing to local or distant cancer relapse, found no predictive value, while non-tumor tissue showed predictive capacity (AUC=0.88) [17]. Cheng et al. reported the biomarkers, including kynurenate, 2-aminobutyrate, succinate, p-cresol, putrescine and fumarate in early diagnosis and stages, which were critical for prognosis and survival [11]. The biomarkers, related to recurrence and prognosis/survival, were shown in the Table 2 and electronic supplementary materials with special markers (Supplementary Tables S2, S3, S4 and S5).

### Altered metabolism in colorectal cancer

#### Cellular respiration/carbohydrate metabolism perturbations

**Figure 3:**
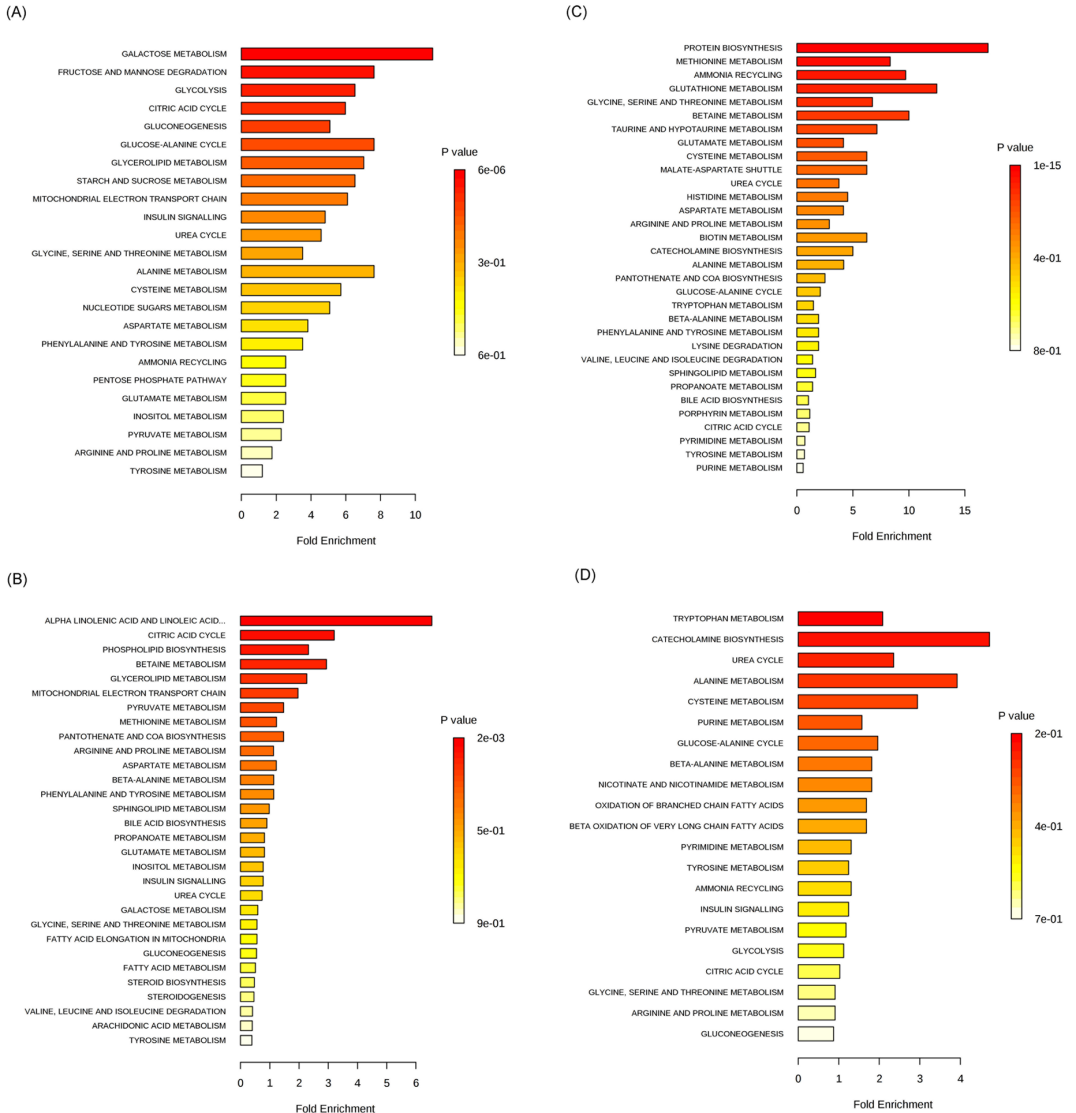
The enriched pathways of metabolites **A**. carbohydrate metabolites. **B**. lipid metabolites. **C**. amino acid metabolites. **D**. nucleotide, ketone, tocopherol and benzoate metabolites. Bar colors indicate different of significance. Bar lengths indicate different fold enrichment.

Altered levels of metabolites, reported in metabolomic studies of CRC related to glycolysis, the TCA cycle and anaerobic respiration, were shown in Supplementary Table S2. Nine metabolite biomarkers, related to above pathways, were reported in more than one metabolomic study, including eight biomarkers which had consistent results and only one biomarker which had contradictory results across different studies. Fumarate, as the TCA intermediate [20], was found decreasing in tissue profiling [19], while elevating in urine profiling[11]. Glucose, as the origin of above pathways, was reported decreasing in six studies, containing four studies on tissue [16, 17, 19, 21], one study on serum [22] and one study on feces specimen [23]. Lactate, a product of anaerobic glycolysis [24], was found increasing in seven studies, including five studies on tissue [13, 16–19] and two studies on serum [14, 25]. Arabitol, galactose, mannose and pyruvate were reported decreasing in all studies, respectively, while glycerol and succinate were found elevating in all studies, respectively. Galactose, galactitol and glucose in perturbed galactose metabolism pathway had the same decreasing trend in all literatures [16, 17, 19, 22, 23], which may be explained by that galactitol and glucose are the products of galactose. The metabolites with the same change tendency in more than one literature had potential clinical significance and were shown in Table 2. All the cellular/carbohydrate metabolites were enriched in twenty-four pathways (Figure 3A).

#### Lipid metabolite perturbations

Metabolites, related to fatty acid oxidation, were frequently altered in CRC patients (Supplementary Table S3). Fifteen biomarkers, related to lipid metabolism pathway, were reported in more than one metabolomic study, including three biomarkers which had contradictory results and twelve biomarkers which had consistent results across the different studies. In one study arachidonic acid was found to be increased in tissue of CRC patients [21] while decreased in another [19]. Fumarate was elevated in urine of CRC cases in one study [11] while decreased in tissue [19]. Increased levels of myristate in tissue of CRC cases [18] was found down-regulated in urine [11]. Lactate, 2-aminobutyrate, choline, hydroxybutyrate, succinate, acetate, oleic acid, glycochenodeoxycholate and phosphocholine (PC) were increased across all studies. Myoinositol, triglycerides and 1-octanol were decreased in all studies. The metabolites with the same change tendency in more than one literature had potential clinical significance and were shown in Table 2. All the lipid metabolites in supplementary table 3 were enriched in thirty pathways (Figure 3B).

#### Amino acid metabolite perturbations

Amino acid metabolism is one of the pathways that had been commonly reported to be altered in CRC in the studies included in this systematic review (Supplementary Table S4). Eighteen biomarkers related to amino acid metabolism pathways were reported in more than one metabolomic study, including eleven contradictory biomarkers and seven consistent biomarkers across different studies. For instance, glycine was reported to be increased in tissues from two studies [16, 19] while to be decreased in serum from two other studies [22, 26]. Alanine was reported to be increased in serum and tissue in two studies [18, 25] while to be decreased in serum and urine in four other studies [11, 14, 26, 27]. Taurine was reported to be increased in tissue in three studies [16, 17, 19] while decreased in the same tissue in another study [13]. Histidine, methionine, and tryptophan were decreased in CRC cases in all studies while glutamic acid, proline/L-proline, iso-glutamine and putrescine were increased in all studies. The metabolites with the same change tendency in more than one literature had potential clinical significance and were shown in Table 2. All the amino acid metabolites were enriched in thirty-two pathways (Figure 3C).

#### Nucleotide metabolites and other significant metabolite perturbations

Nucleotide metabolites and other significant metabolites altered in CRC patients were summarized in Supplementary Table S5. Nine biomarkers were reported in more than one metabolomic study, including five biomarkers which had contradictory results and four biomarkers which had consistent results across different studies. For example, uracil had higher levels in tissues of CRC cases in three studies and in feces in one study [13, 18, 23, 28] while lower in urine in another study [11]. P-cresol was up-regulated in urine of CRC cases in one study [15] while was down-regulated in the same urine in another study [11]. Carnitine and hypoxanthine were reported to be increased in CRC cases in all studies. Phenol and urea were reported to be decreased in CRC cases in all studies. The metabolites with the same change tendency in more than one literature had potential clinical significance and were shown in Table 2. All the metabolites were enriched in twenty-one pathways (Figure 3D).

## DISCUSSION

This systematic review provides a qualitative assessment of studies conducted on metabolomic profiling in CRC. From this review, we found that some individual results were contradicting. For example, Li et al. and Mirnezami et al. found that the glycine was higher in CRC when compared with controls, while Leichtle et al. and Ma et al. found that glycine was lower in CRC. The reason was likely due to different bio-fluids, since Li et al. and Mirnezami et al. performed the metabolomic profiling in the tissues samples, while Leichtle et al. and Ma et al. conducted it in the serum samples [12, 16, 22, 26]. Besides, we have discovered that the diagnostic or predictive accuracy of metabolites were different across studies, and biomarkers for early diagnosis, stage, prognosis, survival and recurrence were distinctive. It could be explained by the diversity of specimens, metabolomic analytical platforms, different experiment subjects and/or sample sizes.

In this review, we presented the diagnostic implications of metabolomic profiling in detection of CRC. Previous studies have reported that the routine noninvasive diagnostic tools in clinical use were not satisfactory [29, 30]. It is known that early diagnosis and detailed stages of CRC have a significant impact on CRC management, prognosis, recurrence, or survival [31–33]. Furthermore, the targeted metabolomic researches certificated that the most results were consistent with the discovery phase[34, 35]. Our results indicated that sample metabolomic profiling could distinguish CRC patients, including early stage patients, from normal controls and will be a promising tool in early noninvasive diagnosis of CRC.

Metabolite perturbations and relevant biological pathways were examined which included cellular respiration, carbohydrate, amino acid, lipid, nucleotide, and ketone metabolisms. There were significant alterations in metabolites of glycolysis, TCA cycle, and anaerobic respiration pathways which indicated significant perturbations of energy metabolism in CRC. Altered energy metabolism, as a hallmark of cancer, was first identified almost a century ago when Warburg discovered that cancer cells primarily used anaerobic glycolysis to produce energy, even in the presence of oxygen, which was called the Warburg effect [36]. Further, the Warburg effect was known to cause an increase in lactate production and lower the pH of malignant tissue, which in turn impaired DNA repair mechanisms [37]. This phenomenon was demonstrated in CRC metabolomics with perturbations of 6-phosphogluconic acid, citrate, formate, isocitrate, pyruvate, 3-phosphoglycerate, L-Glutamine, succinate and lactate in studies. Lipid metabolism also had an essential role in malignant proliferation, suggesting that adipocytes act as an energy source for cancer cells in malignances such as prostate and kidney cancers [38–40]. Increased fatty acid oxidation was associated with an over-expression of uncoupling proteins that could promote chemo resistance in cancer cells through mitochondrial ‘‘uncoupling’’, helping cancer cells to survive [41]. In our systematic review, the fatty acid oxidation alterations included mitochondrial beta-oxidation of long chain saturated fatty acids, oxidation of branched chain fatty acids and mitochondrial beta-oxidation of short chain saturated fatty acids. This phenomenon was demonstrated in CRC metabolomics with perturbations of stearic acid, carnitine, octadecanoic acid and succinate. Consistent with abnormal fatty acid oxidation, abnormal phospholipid biosynthesis were demonstrated in CRC metabolomics with perturbations of phosphocholine, choline, LPA(16:0) and LPC(16:0). As the essential components of biological membranes, abnormal phospholipid biosynthesis in the CRC patients was probably associated with this biological activity and was due to accelerated cell proliferation [42, 43]. Amino acid metabolism was another novel pathway that was commonly altered in cancer cells, including abnormal tryptophan metabolism, abnormal alanine metabolism, abnormal glucose-alanine cycle, abnormal glutamate metabolism, abnormal arginine and proline metabolism, abnormal beta-alanine metabolism, and abnormal histidine metabolism. Nucleotide metabolism was also a novel pathway that was commonly altered in cancer cells, including abnormal thioguanine pathway and abnormal mercaptopurine metabolism pathway.

Overall, metabolomics has revealed multiple dysregulated metabolites that were related to the differences in metabolic pathways between CRC and control samples and potentially could have turned out to be multiple clinically useful biomarkers. Despite the promising preliminary results, a consensus group of biomarkers for CRC has not yet been emerged. The biomarker development in CRC metabolomics has not progressed beyond Phase 1 pre-clinical exploratory studies. Such a group of biomarkers is a necessary prerequisite for larger scale studies of CRC detection. Also, the fusion of metabolic profiling data could enlarge the size of data set and improve the stability of biomarkers detection economically. It is necessary to study effective data fusion method, integrate current data of CRC and re-analyze the fusion data. The standardization of metabolomic platforms, including separating techniques, is crucial to minimize variability due to equipments and approaches to metabolite identification and quantitation. Subsequently, larger studies, addressing a more diverse population, need to be designed and executed. Beyond the question of screening biomarkers, our review provided insights into the biology of CRC development. Apart from the obvious scientific interest, such knowledge will form the basis for new therapeutic interventions that can interrupt these neoplastic pathways. Rigorous adherence to these approaches will set the stage for metabolomics to be validated both as a diagnostic tool and as the basis for a new generation of therapeutic agents for CRC.

## MATERIALS AND METHODS

### Search strategy

A literature search was done through three databases (PubMed, Web of Science and Embase) with the combination of the keywords “metabolomics”, “metabolite”, “metabolome”, “metabolic profiling”, “colorectal cancer”, “colorectal neoplasm”, “colorectal carcinoma”, “colorectal tumor”, “biomarker”, “diagnosis”, “recurrence”, “prognostic” and “survival” in all fields from 1998 to January 2016. Three independent searching procedures were performed according to our aim: diagnosis; prognosis or survival; recurrence. Literature searching for each aim was conducted in three databases, based on search strategy. The inclusions and exclusions were displayed in the section 2.2. After obtaining all papers, we firstly combined literatures according to aims and excluded the duplicates. Then, we screened literatures based on titles and abstracts and excluded articles not meeting our inclusion criteria. Last, we combined all articles and excluded duplicates. All the remaining papers were downloaded in full-text. Two researchers (Zhang Y and Zhao W) independently assessed all articles, based on their full text. When it came to disagreement regarding inclusion or exclusion, they would consult with a senior researcher (Zhang F) and generate a consensus. The searching and screening literature workflow was displayed as follows (see Figure 1).

### Inclusion and exclusion criteria

All studies that investigated the metabolomic profile of biological samples from tissues or bio-fluids of patients with CRC, compared to an appropriate control group, were included in our analysis. We limited our studies to employing mass spectrometry (MS) and nuclear magnetic resonance (NMR). All metabolomic studies concerning human in vitro or animal CRC models were excluded. Only original articles, published in English with full text available, were selected for the final analysis.

### Data extraction and analysis

After we selected the final literature, the following information was extracted from each study, if provided:

1. first author's name and publication year
2. specimen type
3. analytic platform
4. sample size, including number of cases and controls
5. origin
6. whether there was an independent validation
7. whether it was a prospective research
8. significantly altered metabolites in patients with CRC compared to a control group

Data extraction was carried out by two independent researchers (Zhang Y, Zhao W) to avoid author bias.

### Methodological quality assessment

In this study, we applied QUADOMICS, an adaption of quality assessment tool for diagnostic accuracy studies (QUADAS), to assess the methodological quality of the selected studies, which takes into account for the particular challenges when systematic reviews of ‘omics’-based techniques were being performed [44]. The quality of the studies was summarized by the percentage of applied criteria scored positively. We did not use a threshold integer while assessing the quality of studies, as has been previously reported [45]. A cutoff assessing the quality of published studies has not been yet published by either QUADAS or QUADOMICS, as such a cutoff would not sufficiently discriminate between a study with a major methodological flaw that invalidates the results in comparison to one with minor methodological flaws [44, 46, 47]. QUADOMICS can assess the quality of diagnostic studies in a highly dynamic field which faces the challenge of sieving the huge amount of results recently produced [44].

### Metabolites enriched into pathways

The biomarkers extracted from the literatures were enriched into pathways based on cellular/carbohydrate metabolites, lipid metabolites, amino acid metabolites and nucleotide metabolites respectively. The enrichments were performed through MetaboAnalyst software (http://www.metaboanalyst.ca).

## SUPPLEMENTARY MATERIALS FIGURES AND TABLES


