# Supplementary material for: Metabolomics for biomarker discovery in the diagnosis, prognosis, survival and recurrence of colorectal cancer: a systematic review
Source: Oncotarget. 2017 Mar 30;8(21):35460–72. doi: 10.18632/oncotarget.16727 (PMC5471069; doi:10.18632/oncotarget.16727)
Supplement: Supplementary file 1 [file oncotarget-08-35460-s001.docx]

**Supplementary Table 1:QUADOMICS assessment of studies included in the systematic review**

| ITEM | Chan et al., 2009 | Cheng et al., 2012 | Cross et al., 2014 | Dowling et al., 2015 | Ikeda et al., 2012 | Jiménez et al., 2013 | Leichtle et al., 2012 | Li et al., 2013 | Liesenfeld et al., 2015 | Liesenfeld et al., 2015 | Ma et al., 2012 | Manna et al., 2014 | Mirnezami et al., 2014 | Nishiumi et al., 2012 | Phua et al., 2014 | Qiu et al., 2014 | Ritchie et al., 2010 | Silva et al., 2011 | Tan et al., 2013 | Wang et al., 2014 | Wang et al., 2013 | Yue et al., 2013 | Zhu et al., 2014 | YES  % | NO  % | UC  % |
| --- | --- | --- | --- | --- | --- | --- | --- | --- | --- | --- | --- | --- | --- | --- | --- | --- | --- | --- | --- | --- | --- | --- | --- | --- | --- | --- |
| 1 | Y | Y | Y | Y | Y | Y | ? | Y | Y | Y | Y | Y | Y | Y | Y | Y | Y | Y | Y | Y | Y | Y | Y | 96 | 0 | 4 |
| 2 | N | N | N | N | N | N | N | N | N | N | N | N | N | N | N | N | N | N | N | N | N | N | N | 0 | 100 | 0 |
| 3 | Y | Y | ? | Y | Y | Y | Y | Y | Y | Y | Y | Y | Y | Y | Y | Y | Y | Y | Y | Y | Y | Y | Y | 96 | 0 | 4 |
| 4a | Y | Y | Y | Y | Y | Y | Y | Y | Y | Y | Y | Y | Y | Y | Y | Y | Y | ? | Y | Y | Y | Y | Y | 96 | 0 | 4 |
| 4b | ? | Y | Y | ？ | Y | Y | ? | ? | Y | Y | Y | Y | Y | Y | Y | Y | Y | ? | Y | Y | Y | N | Y | 74 | 4 | 22 |
| 5 | Y | Y | Y | Y | Y | Y | Y | Y | Y | Y | Y | Y | Y | Y | Y | Y | Y | Y | Y | Y | Y | Y | Y | 100 | 0 | 0 |
| 6 | Y | Y | Y | Y | Y | Y | ? | Y | Y | Y | ? | ? | Y | Y | Y | Y | Y | Y | Y | Y | Y | ? | Y | 83 | 0 | 17 |
| 7 | Y | Y | Y | Y | Y | Y | Y | Y | Y | Y | ? | Y | Y | Y | Y | ? | Y | Y | Y | Y | Y | ? | ? | 83 | 0 | 17 |
| 8 | Y | Y | Y | Y | Y | Y | Y | Y | Y | Y | ? | Y | Y | Y | Y | ? | Y | ? | ? | Y | Y | ? | ? | 74 | 0 | 26 |
| 9 | Y | Y | Y | Y | Y | Y | Y | Y | Y | Y | ? | Y | Y | Y | ? | ? | Y | ? | Y | Y | Y | ? | ? | 74 | 0 | 26 |
| 10 | Y | Y | Y | Y | Y | Y | Y | Y | Y | Y | Y | Y | Y | Y | Y | Y | Y | Y | Y | Y | Y | Y | Y | 100 | 0 | 0 |
| 11 | Y | Y | Y | Y | Y | Y | Y | Y | Y | Y | ? | Y | Y | Y | Y | ? | Y | ? | ? | Y | Y | ? | ? | 74 | 0 | 26 |
| 12 | N | N | N | N | N | N | N | N | N | N | N | N | N | N | N | N | N | N | N | N | N | N | N | 0 | 100 | 0 |
| 13 | Y | Y | Y | Y | Y | Y | Y | Y | Y | Y | ? | Y | Y | Y | Y | ? | Y | ? | Y | Y | Y | Y | ? | 83 | 0 | 17 |
| 14 | N | N | N | N | N | N | N | N | N | N | N | N | N | N | N | N | N | N | N | N | N | N | N | 0 | 100 | 0 |
| 15 | N | N | N | N | N | N | N | N | N | Y | N | N | Y | Y | N | N | N | N | N | N | N | N | N | 87 | 13 | 0 |
| 16 | Y | Y | N | N | N | Y | N | N | Y | N | N | N | N | Y | Y | Y | Y | N | Y | Y | Y | Y | Y | 57 | 43 | 0 |

**Index:**Item 1 Were selection criteria clearly described? 2. Was the spectrum of patients representative of patients who will receive the test in practice? 3. Was the type of sample fully described? 4. Were the procedures and timing of biological sample collection with respect to clinical factors described with enough detail? 4a. Clinical and physiological factors 4b. Diagnostic and treatment procedures 5. Were handling and pre-analytical procedures reported in sufficient detail and similar for the whole sample? And, if differences in procedures were reported, was their effect on the results assessed? 6. Is the time period between the reference standard and the index test short enough to reasonably guarantee that the target condition did not change between the two tests? 7. Is the reference standard likely to correctly classify the target condition? 8. Did the whole sample or a random selection of the sample receive verification using a reference standard of diagnosis? 9. Did patients receive the same reference standard regardless of the result of the index test? 10. Was the execution of the index test described in sufficient detail to permit replication of the test? 11. Was the execution of the reference standard described in sufficient detail to permit its replication? 12. Were the index test results interpreted without knowledge of the results of the reference standard? 13. Were the reference standard results interpreted without knowledge of the results of the index test? 14. Were the same clinical data available when test results were interpreted as would be available when the test is used in practice? 15. Were interpretable / intermediate test results reported? 16. Is it likely that the presence of over-fitting was avoided; Y=criteria achieved, N=criteria not achieved, ?=Unclear

**Supplementary Table 2:Altered cellular / carbohydrate metabolites in biological samples of colorectal cancer**

| METABOLITE | 1-Deoxyglucose | 6-Phosphogluconic acid | Arabitol | Fructose | Fucose | Galactitol | Galactose | Glucose | Glucuronate | Mannose | Sorbose | Threonate | Xylose | 3-Phosphoglycerate | Glycerol | Ribitol | lacate | fumarate | malate | succinate | pyruvate |
| --- | --- | --- | --- | --- | --- | --- | --- | --- | --- | --- | --- | --- | --- | --- | --- | --- | --- | --- | --- | --- | --- |
| Chan et al., 2009⊕ |  |  |  |  |  |  | **↓** | **↓** |  | **↓** |  |  |  |  | **↑** |  | **↑** | **↓** | **↓** |  |  |
| Cheng et al., 2012※ |  |  | **↓** |  |  |  |  |  | **↓** |  | **↓** | **↓** | **↓** |  |  |  |  | **↑** |  | **↑** | **↓** |
| Ikeda et al., 2012＃ |  |  |  |  |  |  |  |  |  |  |  |  |  |  |  |  | **↑** |  |  |  |  |
| Jimenez et al., 2013⊕ |  |  |  |  |  |  |  | **↓▲** |  |  |  |  |  |  |  |  | **↑** |  |  |  |  |
| Liesenfeld et al., 2015⊕ |  |  |  |  | **↓** | **↓** |  | **↓** |  | **↓** |  |  |  |  |  |  |  |  |  |  |  |
| Liesenfeld et al., 2015※ |  |  | **↓** |  |  |  |  |  |  |  |  |  |  |  |  |  |  |  |  |  |  |
| Ma et al., 2012＃ | **↓** |  |  |  |  |  |  | **↓** |  |  |  |  |  |  |  | **↓** |  |  |  |  |  |
| Mirnezami et al., 2014⊕ |  |  |  |  |  |  |  | **↓** |  |  |  |  |  |  |  |  | **↑** |  |  | **▲** |  |
| Phua et al., 2014⊙ |  |  |  | **↓** |  |  | **↓** | **↓** |  |  |  |  |  | **↓** |  |  |  |  |  |  |  |
| Qiu et al., 2014⊕ |  |  |  |  |  |  |  |  |  |  |  |  |  |  | **↑◆** |  | **↑** |  |  |  |  |
| Tan et al., 2013＃ |  | **↓** |  |  |  |  |  |  |  |  |  |  |  |  |  |  |  |  |  |  |  |
| Wang et al., 2013⊕ |  |  |  |  |  |  |  |  |  |  |  |  |  |  |  |  | **↑** |  |  | **↑** |  |
| Zhu et al., 2014＃ |  |  |  |  |  |  |  |  |  |  |  |  |  |  |  |  | **↑** |  |  |  | **↓** |

Note: ↑/↓=upregulated / downregulated in CRC, ▲=stage related biomarker, ◆=recurrence related biomarker； ＃= plasma/serum specimen, ※= urine specimen, ⊕= tissue specimen, ⊙=feces specimen

**Supplementary Table 3:Altered lipid metabolites in biological samples of colorectal cancer**

| METABOLITE | Hexadecanedioic acid | Malonicacid/3-hydroxybutyrate (3HBA) | Dihydrosphingosine | Eicosatrienoic acid | GPC | Linoleic acid | LPC(18:1) | LPC(18:2) | LPC(20:4) | LPC(22:6) | Maleic acid | Pantothenate | Phosphocreatine | Phosphoenolpyruvate (PEP) | 2-Aminobutyrate | 3-Hydroxyisobutyric acid | 3-Hydroxypropionic acid | Choline | Glycolic acid | Hydroxybutyrate | Iso-butyrate | Lactic acid | Sphinganine | Succinate | Thiodiglycolic acid | Trans-2-dodecen-1-ol | Triglycerides | Cyclooctylmethanol | 11,14-Eicosadienoic acid | 11-Eicosenoic acid | 1-Hexadecanol | 1-Monooleoylglycerol | Ceramides | 1-O-Heptadecylglycerol | 2-Hydroxyestradiol | Acetate | Aconitate | Arachidonic acid | Cholesterol | Citrate | Formate | | Fumarate | 3-Hydroxybutyric | Glycochenodeoxycholate |
| --- | --- | --- | --- | --- | --- | --- | --- | --- | --- | --- | --- | --- | --- | --- | --- | --- | --- | --- | --- | --- | --- | --- | --- | --- | --- | --- | --- | --- | --- | --- | --- | --- | --- | --- | --- | --- | --- | --- | --- | --- | --- | --- | --- | --- | --- |
| Chan et al., 2009⊕ |  |  |  |  |  |  |  |  |  |  |  |  |  |  |  |  |  | **↑** |  |  |  |  |  |  |  |  |  |  | **↑** | **↑** | **↓** | **↑** |  | **↑** |  |  |  | **↓** | **↑** |  |  | | **↓** |  |  |
| Cheng et al., 2012※ |  |  |  |  |  |  |  |  |  |  |  |  |  |  | **↑▲** |  |  |  |  | **↑** |  |  |  | **↑▲** |  |  |  |  |  |  |  |  |  |  | **↓** |  | **↓** |  |  | **↓** |  | | **↑▲** |  |  |
| Cross et al., 2014＃ |  |  |  |  |  |  |  |  |  |  |  |  |  |  |  |  |  |  |  |  |  |  |  |  |  |  |  |  |  |  |  |  |  |  |  |  |  |  |  |  |  | |  |  | **↑** |
| Ikeda et al., 2012＃ |  |  |  |  |  |  |  |  |  |  |  |  |  |  |  | **↑** | **↑** |  | **↑** |  |  | **↑** |  |  | **↑** |  |  |  |  |  |  |  |  |  |  |  |  |  |  |  |  | |  |  |  |
| Jimenez et al., 2013⊕ |  |  |  |  |  |  |  |  |  |  |  |  |  |  |  |  |  | **↑●** |  |  | **↑●◆** |  |  |  |  |  | **↓▲** |  |  |  |  |  |  |  |  | **↑◆** |  |  |  |  |  |  | |  |  |
| Li et al., 2013＃ | **↓★** |  |  | **↓** |  |  | **↑** | **↑★** | **↑★** | **↑★** |  |  |  |  |  |  |  |  |  |  |  |  |  |  |  |  |  |  |  |  |  |  |  |  |  |  |  |  |  |  |  | |  |  |  |
| Liesenfeld et al., 2015  ⊕ |  |  |  |  |  |  |  |  |  |  |  |  |  |  |  |  |  |  |  |  |  |  |  |  |  |  |  |  |  |  |  |  | **↑** |  |  |  |  | **↑** |  |  |  | |  |  |  |
| Ma et al., 2012＃ |  |  |  |  |  |  |  |  |  |  |  |  |  |  |  |  |  |  |  |  |  |  |  |  |  |  |  |  |  |  |  |  |  |  |  |  |  |  |  |  |  | |  | **↑** |  |
| Mirnezami et al., 2014⊕ |  |  |  |  | **↑▲** |  |  |  |  |  |  |  |  |  |  |  |  |  |  |  |  |  |  |  |  |  | **↓▲** |  |  |  |  |  |  |  |  | **▲** |  |  |  |  |  | |  |  |  |
| Nishiumi et al., 2012＃ |  |  |  |  |  |  |  |  |  |  |  |  |  |  |  |  |  |  |  | **↑** |  |  |  |  |  |  |  |  |  |  |  |  |  |  |  |  |  |  |  |  |  | |  |  |  |
| Phua et al., 2014⊙ |  |  |  |  |  | **↓** |  |  |  |  |  |  |  |  |  |  |  |  |  |  |  |  |  |  |  |  |  |  |  |  |  |  |  |  |  |  |  |  |  |  |  | |  |  |  |
| Qiu et al., 2014⊕ |  |  |  |  |  |  |  |  |  |  |  |  |  |  | **↑◆** |  |  |  |  |  |  |  |  |  |  |  |  |  |  |  |  |  |  |  |  |  |  |  |  |  |  | |  |  |  |
| Wang et al., 2014◎ |  |  |  |  |  |  |  |  |  |  |  |  |  |  |  |  |  |  |  |  |  |  |  |  |  | **↑** |  | **↑** |  |  |  |  |  |  |  |  |  |  |  |  |  | |  |  |  |
| Wang et al., 2013⊕ |  |  |  |  |  |  |  |  |  |  |  |  | **↓** |  |  |  |  |  |  |  |  |  |  | **↑** |  |  |  |  |  |  |  |  |  |  |  | **↑** |  |  |  |  | **↑** | |  |  |  |
| Yue et al., 2013※ |  |  | **↓** |  |  |  |  |  |  |  |  |  |  |  |  |  |  |  |  |  |  |  | **↓** |  |  |  |  |  |  |  |  |  |  |  |  |  |  |  |  |  |  | |  |  |  |
| Zhu et al., 2014＃ |  | **↓** |  |  |  |  |  |  |  |  | **↑** | **↑** |  | **↓** |  |  |  |  |  |  |  |  |  |  |  |  |  |  |  |  |  |  |  |  |  |  |  |  |  |  |  | |  |  | **↑** |

**Supplementary Table 3 Continued**

| METABOLITE | 2-Hydroxyglutarate | 2-Hyd-roxybutyric acid | 3-(3-Hydroxyphenyl)-3-hydroxypropionate | Betaine | 1-Octanol | LPA(16:0) | LPA(18:0) | LPC(16:0) | LPC(18:0) | Cis-aconitic acid | Glycocholate | Hydroxyacetate | Hydroxycotinine | Isocitrate | Lysoglycerophospholipids | Malate | Myoinositol | Myristate | Oleamide | Oleate(18:1n9) | Palmitic acid | P-Cymene | Phosphate | Phosphatidylcholines | Phosphatidylethanolamines | Phosphocholine (PC) | Phosphoethanolamine (PE) | Plasmenyl-phospholipids | Polyethylene glycol (PEG) | Propyl octadecanoate | Scyllo-inositol | Select phosphatidylethanolamines | γ-Terpinene | Sphingomyelin lipids | Stearate(18:0) | Stearic acid | Lactate | Linolenic acid | Octadecanoic acid | O-octanoyl-R-carnitine | Oleic acid | Palmitic amide | Palmitoleate | Indole-acetate | LPC |
| --- | --- | --- | --- | --- | --- | --- | --- | --- | --- | --- | --- | --- | --- | --- | --- | --- | --- | --- | --- | --- | --- | --- | --- | --- | --- | --- | --- | --- | --- | --- | --- | --- | --- | --- | --- | --- | --- | --- | --- | --- | --- | --- | --- | --- | --- |
| Chan et al., 2009⊕ |  |  |  |  |  |  |  |  |  |  |  |  |  |  |  | **↓** |  |  |  |  | **↑** |  | **↑** |  |  | **↑** | **↑** |  | **↓** | **↑** | **↑** |  |  |  |  | **↑** | **↑** |  |  |  | **↑** |  |  |  |  |
| Cheng et al., 2012※ |  |  |  |  |  |  |  |  |  |  |  | **↓** |  | **↓** |  |  |  | **↓** |  |  |  |  |  |  |  |  |  |  |  |  |  |  |  |  |  |  |  |  |  |  |  |  |  | **↓** |  |
| Cross et al., 2014＃ |  |  |  |  |  |  |  |  |  |  |  |  | **↑** |  |  |  |  |  |  |  |  |  |  |  |  |  |  |  |  |  |  |  |  |  |  |  |  |  |  |  |  |  |  |  |  |
| Dowling et al., 2015＃ |  |  |  |  |  |  |  |  |  |  |  |  |  |  |  |  |  |  |  | **↓** |  |  |  |  |  |  |  |  |  |  |  |  |  |  | **↓** |  |  |  |  |  |  |  |  |  |  |
| Jimenez et al., 2013⊕ |  |  |  |  |  |  |  |  |  |  |  |  |  |  |  |  |  |  |  |  |  |  |  |  |  |  |  |  |  |  |  |  |  |  |  |  | **↑** |  |  |  |  |  |  |  |  |
| Li et al., 2013＃ |  |  |  |  |  | **↑** | **↑** | **↑****★** | **↑** |  |  |  |  |  |  |  |  |  | **↓** |  | **★** |  |  |  |  |  |  |  |  |  |  |  |  |  |  |  |  |  | **↓★** |  |  | **↓★** |  |  |  |
| Liesenfeld et al., 2015⊕ |  |  |  |  |  |  |  |  |  |  |  |  |  |  | **↑** |  |  |  |  |  |  |  |  | **↑** | **↑** | **↑** |  | **↓** |  |  |  | **↓** |  | **↓** |  |  |  |  |  |  |  |  |  |  | **↑** |
| Liesenfeld et al., 2015※ | **↑** |  | **↑** |  |  |  |  |  |  | **↓** |  |  |  |  |  |  | **↓** |  |  |  |  |  |  |  |  |  |  |  |  |  |  |  |  |  |  |  |  |  |  |  |  |  |  |  |  |
| Manna et al., 2014⊕ |  |  |  | **↑** |  |  |  |  |  |  |  |  |  |  |  |  |  |  |  |  |  |  |  |  |  |  |  |  |  |  |  |  |  |  |  |  |  |  |  |  |  |  |  |  |  |
| Mirnezami et al., 2014⊕ |  |  |  |  |  |  |  |  |  |  |  |  |  |  |  |  |  |  |  |  |  |  |  |  |  |  |  |  |  |  |  |  |  |  |  |  | **↑** |  |  |  |  |  |  |  |  |
| Qiu et al., 2014⊕ |  |  |  |  |  |  |  |  |  |  |  |  |  |  |  |  | **↓◆** | **↑◆** |  |  |  |  |  |  |  |  |  |  |  |  |  |  |  |  |  |  | **↑◆** |  |  |  |  |  | **↑◆** |  |  |
| Silva et al., 2011※ |  |  |  |  | **↓** |  |  |  |  |  |  |  |  |  |  |  |  |  |  |  |  | **↑** |  |  |  |  |  |  |  |  |  |  | **↑** |  |  |  |  |  |  |  |  |  |  |  |  |
| Tan et al., 2013＃ |  | **↑** |  |  |  |  |  |  |  |  |  |  |  |  |  |  |  |  |  |  |  |  |  |  |  |  |  |  |  |  |  |  |  |  |  |  |  |  |  |  | **↑** |  |  |  |  |
| Wang et al., 2013⊕ |  |  |  |  | **↓** |  |  |  |  |  |  |  |  |  |  |  | **↓** |  |  |  |  |  |  |  |  |  |  |  |  |  |  |  |  |  |  |  | **↑** |  |  |  |  |  |  |  |  |
| Yue et al., 2013※ |  |  |  |  |  |  |  |  |  |  |  |  |  |  |  |  |  |  |  |  |  |  |  |  |  |  |  |  |  |  |  |  |  |  |  |  |  |  |  | **↓** |  |  |  |  |  |
| Zhu et al., 2014＃ |  |  |  |  |  |  |  |  |  |  | **↑** |  |  |  |  |  |  |  |  |  |  |  |  |  |  |  |  |  |  |  |  |  |  |  |  |  |  | **↓** |  |  |  |  |  |  |  |

Note: ↑/↓= upregulated / downregulated in CRC, ▲=stage related biomarker, **★**=early diagnosis related biomarker, ◆=recurrence related biomarker, ●=prognosis/survival related biomarker; ＃= plasma/serum specimen, ※= urine specimen, ⊕= tissue specimen, ⊙=feces specimen, ◎= exhaled breath

**Supplementary Table 4: Altered amino acid metabolites in biological samples of colorectal cancer**

| METABOLITE | 5-Oxoproline | Aspartate | Asymmetric-dimethylarginine | Glycine | cysteine | Glutamic acid | Glutathione | Hydroxyproline/aminolevulinate2-aminoadipate | Hydroxyproline/aminolevulinate | N1-Acetylspermidine | Putrescine | S-Adenosylhomocysteine | S-Adenosylmethionine | Sarcosine | Serine | Symmetric-dimethylarginine | 5-Hydroxy-tryptophan | Alanine/ L-Alanine/β-Alanine | Asparagine | Aspartic acid | Creatine | Histidine | Taurine | Tyrosine | Valine | Valine-metabolites | Iso-glutamine | Lysine |
| --- | --- | --- | --- | --- | --- | --- | --- | --- | --- | --- | --- | --- | --- | --- | --- | --- | --- | --- | --- | --- | --- | --- | --- | --- | --- | --- | --- | --- |
| Chan et al., 2009⊕ |  |  |  | **↑** |  |  |  |  |  |  |  |  |  |  |  |  |  |  |  |  |  |  | **↑** |  |  |  |  |  |
| Cheng et al., 2012※ |  |  |  |  |  |  |  |  |  |  | **↑▲** |  |  |  |  |  | **↓** | **↓** |  |  |  |  |  | **↓** |  |  |  |  |
| Ikeda et al., 2012＃ |  |  |  |  |  | **↑** |  |  |  |  |  |  |  |  |  |  |  | **↑** | **↑** |  |  |  |  |  |  |  |  |  |
| Jimenez et al., 2013⊕ |  |  |  |  |  |  |  |  |  |  |  |  |  |  |  |  |  |  |  |  |  |  | **↑▲** | **↑▲** |  |  | **↑** |  |
| Leichtle et al., 2012＃ |  |  |  | **↓** |  |  |  |  |  |  |  |  |  | **↓** |  |  |  | **↓** |  | **↓** |  | **↓** |  | **↓** | **↓** |  |  | **↓** |
| Liesenfeld et al., 2015※ |  |  |  |  |  |  |  |  |  |  |  |  |  |  |  |  |  |  | **↓** |  |  |  |  |  |  | **↑** |  |  |
| Ma et al., 2012＃ |  |  |  | **↓** |  |  |  |  |  |  |  |  |  |  |  |  |  |  |  |  |  |  |  |  |  |  |  |  |
| Manna et al., 2014⊕ |  |  | **↑** |  |  | **↑** |  |  |  | **↑** |  | **↑** | **↑** |  |  | **↑** |  |  |  | **↑** |  |  |  |  |  |  |  | **↑** |
| Mirnezami et al., 2014⊕ |  |  |  | **↑** |  |  |  |  |  |  |  |  |  |  |  |  |  |  |  |  |  |  | **↑** |  |  |  | **↑** |  |
| Qiu et al., 2014⊕ | **↑◆** | **↑◆** |  |  | **↑◆** |  |  |  |  |  | **↑◆** |  |  |  |  |  |  | **↑◆** |  |  |  |  |  |  |  |  |  |  |
| Tan et al., 2013＃ |  |  |  |  |  |  |  |  |  |  |  |  |  |  |  |  |  | **↓** |  |  |  | **↓** |  |  |  |  |  |  |
| Wang et al., 2013⊕ |  |  |  |  |  |  | **↑** |  |  |  |  |  |  |  | **↑** |  |  |  |  |  | **↓** |  | **↓** | **↑** |  |  |  | **↑** |
| Zhu et al., 2014＃ |  |  |  |  |  |  |  | **↑** | **↑** |  |  |  |  |  |  |  |  | **↓** |  |  |  | **↓** |  |  |  |  |  |  |

**Supplementary Table 4 Continued**

| METABOLITE | Methionine | Gamma-glutamylated amino acid | Proline/ L-Proline | Phenylalanine /L-Phenylalanine | N-Acetyl-L-lysine | dimethylglycine | Gamma-glutamylleucine | Gammaglutamylvaline | Glutamate | Glutamate?pyroglutamate | Citric acid | Threonine | Trimethylamine N-oxide | Leucine | L-Glutamic acid | L-Glutamine | L-Methionine | Tryptophan | L-threonine | Leucic | leucic acid | L-valine | N-Acetylglycine |
| --- | --- | --- | --- | --- | --- | --- | --- | --- | --- | --- | --- | --- | --- | --- | --- | --- | --- | --- | --- | --- | --- | --- | --- |
| Chan et al., 2009⊕ |  |  | **↑** | **↑** |  |  |  |  |  |  |  |  |  |  |  |  |  |  |  |  |  |  |  |
| Cheng et al., 2012※ |  |  |  |  | **↓** |  |  |  |  |  |  |  | **↓** |  |  |  |  | **↓** |  |  |  |  |  |
| Dowling et al., 2015＃ |  | **↓** |  |  |  |  | **↓** | **↓** |  |  |  |  |  |  |  |  |  |  |  |  |  |  |  |
| Ikeda et al., 2012＃ |  |  | **↑** |  |  |  |  |  |  |  |  |  |  |  | **↑** | **↑** | **↑** |  |  |  |  |  |  |
| Jimenez et al., 2013⊕ |  |  |  | **↑▲** |  |  |  |  |  |  |  |  |  |  |  |  |  |  |  |  |  |  |  |
| Leichtle et al., 2012＃ | **↓** |  |  |  |  |  |  |  |  |  |  | **↓** |  | **↓** |  |  |  |  |  |  |  |  |  |
| Liesenfeld et al., 2015※ |  |  | **↑▲●** | **↓** |  |  |  |  |  | **↓** |  |  |  |  |  |  |  |  |  |  |  |  |  |
| Ma et al., 2012＃ |  |  |  |  |  |  |  |  |  |  |  |  |  |  |  |  |  |  | **↓** |  |  | **↓** |  |
| Manna et al., 2014⊕ |  |  | **↑** |  |  | **↑** |  |  |  |  |  |  |  |  |  |  |  |  |  |  |  |  |  |
| Phua et al., 2014⊙ |  |  | **↑** |  |  |  |  |  |  |  | **↓** |  |  |  |  |  |  |  |  |  |  |  |  |
| Qiu et al., 2014⊕ |  |  |  |  |  |  |  |  | **↑◆** |  |  |  |  |  |  |  |  |  |  |  |  |  |  |
| Tan et al., 2013＃ | **↓** |  |  | **↓** |  |  |  |  | **↓** |  |  |  |  |  |  |  |  | **↓** |  |  |  |  |  |
| Wang et al., 2013⊕ |  |  |  |  |  | **↓** |  |  |  |  |  | **↑** |  |  |  |  |  |  |  |  |  |  |  |
| Zhu et al., 2014＃ | **↓** |  |  |  |  | **↓** |  |  |  |  |  |  |  |  |  |  |  |  |  | **↑** | **↑** |  | **↓** |

Note: ↑/↓=upregulated / downregulated in CRC, ▲=stage related biomarker, ◆=recurrence related biomarker, ●=prognosis/survival related biomarker；

＃= plasma/serum specimen, ※= urine specimen, ⊕= tissue specimen, ⊙=feces specimen, ◎= exhaled breath

**Supplementary Table 5:Altered nucleotide, ketone, tocopherol and benzoate metabolites in biological samples of colorectal cancer**

| METABOLITE | Inosine | Hippuric acid | Homovanillate | P-cresol | Phenol | Pyrogallol | C16H22O4 | Dodecane | Ethylaniline | Glucuronic lactone | Isogluamine | Kynurenine | Kyrunine | Carnitine | Nicotinic acid | Cyclohexanone | Xanthine | Scyllo-inositol | 1,2,4-Trimethylbenzene | 1,2-Dihydro-1,1,6-trimethyl-naphthalene | 1,4,5-Trimethyl-naphthalene | 2-Hydroxyhippurate | 3-Hydroxyhippurate | 4-Hydroxyhippurate | 2,7-Dimethyl-quinoline | 2-Methoxythiophene | 2-Methyl-3-phenyl-2-propenal | 3-Indoxylsulfate | 4-Aminohippurate | 4-Methyl-phenol | Bornylene | Creatinine | Dimethyl disulphide | Heptanal | 3-hydroxy-2,4,4-trimethylpentyl 2-methylpropanoate |
| --- | --- | --- | --- | --- | --- | --- | --- | --- | --- | --- | --- | --- | --- | --- | --- | --- | --- | --- | --- | --- | --- | --- | --- | --- | --- | --- | --- | --- | --- | --- | --- | --- | --- | --- | --- |
| Chan et al., 2009⊕ |  |  |  |  |  |  |  |  |  |  |  |  |  |  |  |  |  | **↑** |  |  |  |  |  |  |  |  |  |  |  |  |  |  |  |  |  |
| Cheng et al., 2012※ |  |  | **↓** | **↓▲** | **↓** |  |  |  |  |  |  |  |  |  |  |  |  |  |  |  |  |  |  |  |  |  |  |  | **↓** |  |  | **↓** |  |  |  |
| Ikeda et al., 2012＃ |  |  |  |  |  |  |  |  |  | **↑** |  |  |  |  |  |  |  |  |  |  |  |  |  |  |  |  |  |  |  |  |  |  |  |  |  |
| Liesenfeld et al., 2015※ |  |  |  | **↑** |  | **↓** |  |  |  |  |  |  |  |  |  |  |  |  |  |  |  | **↓** | **↓** | **↓** |  |  |  | **↑** |  |  |  |  |  |  |  |
| Manna et al., 2014⊕ |  |  |  |  |  |  |  |  |  |  |  |  |  | **↑** |  |  | **↑** |  |  |  |  |  |  |  |  |  |  |  |  |  |  |  |  |  |  |
| Mirnezami et al., 2014⊕ |  |  |  |  |  |  |  |  |  |  |  |  |  |  |  |  |  | **↑** |  |  |  |  |  |  |  |  |  |  |  |  |  |  |  |  |  |
| Nishiumi et al., 2012＃ |  |  |  |  |  |  |  |  |  |  |  | **↑** |  |  |  |  |  |  |  |  |  |  |  |  |  |  |  |  |  |  |  |  |  |  |  |
| Phua et al., 2014⊙ | **↓** |  |  |  |  |  |  |  |  |  |  |  |  |  | **↓** |  |  |  |  |  |  |  |  |  |  |  |  |  |  |  |  | **↓** |  |  |  |
| Qiu et al., 2014⊕ |  |  |  |  |  |  |  |  |  |  |  |  | **↑◆** |  |  |  |  |  |  |  |  |  |  |  |  |  |  |  |  |  |  |  |  |  |  |
| Silva et al., 2011※ |  |  |  |  |  |  |  |  |  |  |  |  |  |  |  |  |  |  | **↑** | **↑** | **↑** |  |  |  | **↑** | **↑** | **↑** |  |  | **↑** | **↑** |  | **↓** | **↓** |  |
| Tan et al., 2013＃ |  |  |  |  | **↓** |  |  |  |  |  |  |  |  | **↑** |  |  |  |  |  |  |  |  |  |  |  |  |  |  |  |  |  | **↑** |  |  |  |
| Wang et al., 2014◎ |  |  |  |  |  |  |  | **↑** | **↑** |  |  |  |  |  |  | **↑** |  |  |  |  |  |  |  |  |  |  |  |  |  |  |  |  |  |  | **↑** |
| Yue et al., 2013※ |  |  |  |  |  |  | **↓** |  |  |  |  |  |  |  |  |  |  |  |  |  |  |  |  |  |  |  |  |  |  |  |  |  |  |  |  |
| Zhu et al., 2014＃ |  | **↑** |  |  |  |  |  |  |  |  |  |  |  |  |  |  |  |  |  |  |  |  |  |  |  |  |  |  |  |  |  |  |  |  |  |

**Supplementary Table 5 Continued**

| METABOLITE | hydroxyl hippurate | Anisole | Acetylcarnitine | 2-Methoxyphenol | Dopamine | Guajacol | Hippurate | Hexanal | Hypoxanthine | Indole | Indoleacetate | Kynurenate | Maleamate,hydroquinone | Pyruvate | Serotonin(5-HT) | Uracil | Urea | Uridine | P-cresol-b-Oglucuronide | Pyridoxal (Vitamin B6) | 2,2-dimethyldecane | 2,3-Butanediol |
| --- | --- | --- | --- | --- | --- | --- | --- | --- | --- | --- | --- | --- | --- | --- | --- | --- | --- | --- | --- | --- | --- | --- |
| Chan et al., 2009⊕ |  |  |  |  |  |  |  |  |  |  |  |  |  |  |  |  |  | **↑** |  |  |  |  |
| Cheng et al., 2012※ |  |  | **↑** |  |  |  | **↓** |  |  | **↓** | **↓** | **↓▲** |  | **↓** |  | **↓** | **↓** | **↓** |  | **↓** |  |  |
| Dowling et al., 2015＃ |  |  |  |  |  |  |  |  |  |  |  |  |  |  | **↑** |  |  |  |  |  |  |  |
| Liesenfeld et al., 2015※ | **↓** |  |  |  | **↓** | **↓** |  |  |  |  |  |  | **↓** |  |  |  |  |  | **↑▲●** |  |  | **↓** |
| Manna et al., 2014⊕ |  |  |  |  |  |  |  |  | **↑** |  |  |  |  |  |  | **↑** |  |  |  |  |  |  |
| Phua et al., 2014⊙ |  |  |  |  |  |  |  |  |  |  |  |  |  |  |  | **↑** |  | **↑** |  |  |  |  |
| Qiu et al., 2014⊕ |  |  |  |  |  |  |  |  | **↑◆** |  |  |  |  |  |  | **↑◆** |  |  |  |  |  |  |
| Silva et al., 2011※ |  | **↑** |  | **↑** |  |  |  | **↓** |  |  |  |  |  |  |  |  |  |  |  |  |  |  |
| Tan et al., 2013＃ |  |  |  |  |  |  |  |  |  |  |  |  |  | **↑** |  |  | **↓** |  |  |  |  |  |
| Wang et al., 2014◎ |  |  |  |  |  |  |  |  |  |  |  |  |  |  |  |  |  |  |  |  | **↑** |  |
| Wang et al., 2013⊕ |  |  |  |  |  |  |  |  |  |  |  |  |  |  |  | **↑** |  |  |  |  |  |  |
| Zhu et al., 2014＃ |  |  |  |  |  |  |  |  |  |  |  |  |  | **↑** |  |  |  |  |  |  |  |  |

Note: ↑/↓=upregulated / downregulated in CRC, ▲=stage related biomarker, ◆=recurrence related biomarker, ●=prognosis/survival related biomarker ;

＃= plasma/serum specimen, ※= urine specimen, ⊕= tissue specimen, ⊙=feces specimen, ◎= exhaled breath
